# Mineralization of Acephate, a Recalcitrant Organophosphate Insecticide Is Initiated by a Pseudomonad in Environmental Samples

**DOI:** 10.1371/journal.pone.0031963

**Published:** 2012-04-04

**Authors:** Aleem Basha Pinjari, Boris Novikov, Yohannes H. Rezenom, David H. Russell, Melinda E. Wales, Dayananda Siddavattam

**Affiliations:** 1 Department of Animal Sciences, School of Life Sciences, University of Hyderabad, Gachibowli, Hyderabad, India; 2 Department of Biochemistry and Biophysics, Texas A&M University, College Station, Texas, United States of America; 3 Department of Chemistry, Texas A&M University, College Station, Texas, United States of America; J. Craig Venter Institute, United States of America

## Abstract

An aerobic bacterium capable of breaking down the pesticide acephate (*O,S*-dimethyl acetyl phosphoramidothioic acid) was isolated from activated sludge collected from a pesticide manufacturing facility. A phylogenetic tree based on the 16 S rRNA gene sequence determined that the isolate lies within the Pseudomonads. The isolate was able to grow in the presence of acephate at concentrations up to 80 mM, with maximum growth at 40 mM. HPLC and LC-MS/MS analysis of spent medium from growth experiments and a resting cell assay detected the accumulation of methamidophos and acetate, suggesting initial hydrolysis of the amide linkage found between these two moieties. As expected, the rapid decline in acephate was coincident with the accumulation of methamidophos. Methamidophos concentrations were maintained over a period of days, without evidence of further metabolism or cell growth by the cultures. Considering this limitation, strains such as described in this work can promote the first step of acephate mineralization in soil microbial communities.

## Introduction

Acephate belongs to a large group of organophosphorus pesticides, known to be inhibitors of acetylcholinesterase activity, which have been extensively used in world agriculture to control insect pests of a number of economically important crops. Organophosphates (OPs) were developed as replacements for the more persistent organochlorines [1], [2]. Although OPs are less persistent in the environment, extensive and indiscriminate use has led to the accumulation of their residues in various components of the environment [3]. The reported cases of poisoning in the United States decreased after the U.S. Environmental Protection Agency implemented the Food Quality Protection Act of 1996, which prohibited the use of organophosphate insecticides in residential environments [4], [5]. In spite of this, thousands of cases are reported every year in the United States [6], [7] and around the world [8], [9], due to accidental release, intentional self-poisoning or to consumption of contaminated food [10]. Acephate is believed to be a relatively safe pesticide for humans, acting only as a weak inhibitor of AChE. This toxicity is restricted by a requirement for metabolic conversion to methamidophos and by methamidophos inhibition of the mammal carboxyamidase, which promotes this conversion [11]. In spite of this, many reported observations indicate that the effects of pesticide exposure, including acephate, are not restricted to anticholinesterase action [12], [13], [14], [15], [16], but include genotoxicity [17] and teratogenicity [18]. Such serious health consequences signify a requirement for a better understanding of the fate of acephate in the environment, and the development of safe, reliable and eco-friendly technologies for the elimination of acephate and other OP compounds from contaminated areas.

Although acephate usually degrades in soils, ground water and plants with a half-life of 3–6 days, in some soils the half-life may be increased to more than 13 days [3], [19], [20], [21]. The degradation of acephate has been demonstrated to be promoted by microorganisms by an order of magnitude increase in stability of acephate in sterilized soil [22]. Temperature and pH also contribute to its persistence, demonstrated by a half-life increase from 10 days to 1 year in water at pH 6.0 and 5°C [23]. In frozen fruit samples, acephate may be stable up to 14 months [24]. It is clear that, under favourable conditions, acephate can persist in the environment and food for significant periods of time.

**Figure 1:**
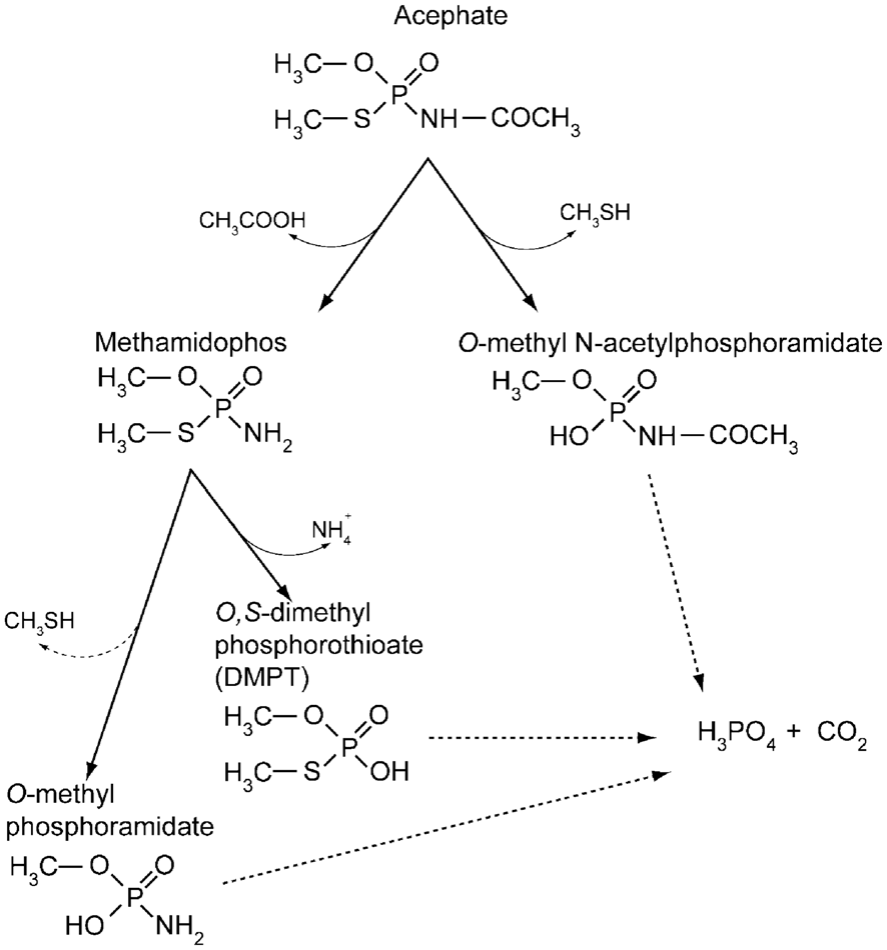
Generalized acephate degradation pathways in *soil (FAO, 2004).*

It has been proposed that acephate in the environment can be mineralized to CO_2_, methyl mercaptan and phosphoric acid through either methamidophos or *O*-methyl N-acetylphosphoramidate, *O,S*-dimethyl phosphorothioate (DMPT) and further to CO_2_, methyl mercaptan and phosphoric acid (Fig. 1) [25]. Little is known about the microorganisms or enzymes responsible for each step of those pathways. Although studies on acephate degradation in soils have been reported during the last decade [21], [22], [26], only one has identified a bacterium that can initiate the pathway by hydrolysis of acephate. In this study, which was focused on methamidophos degradation, it was shown that *Hyphomicrobium* sp. MAP1 can degrade several OP compounds, including acephate. No analysis of degradation products was reported [27]. In the present study, the isolation and characterization of a bacterial strain from the activated sludge of a pesticide-manufacturing unit is reported. This bacterium, designated as *Pseudomonas* sp. Ind01, uses acephate as a source of carbon to support cell growth and can promote the first step of acephate mineralization in soil microbial communities.

## Results

### Strain Ind01 is a Pseudomonad

A Gram negative, rod-shaped bacterium that can utilize acephate as a sole source of carbon was isolated from the activated sludge collected from a pesticide manufacturing plant. The isolate, designated Ind01, was chloramphenicol and ampicillin resistant, but sensitive to kanamycin. Cells are 0.5×1.3 µm in size and possess a single terminal flagellum (Fig. S1).

The sequence of the 16 S rRNA gene of the isolate was determined and used to construct a phylogram (Fig. S2). This analysis placed the Ind01 isolate into the genus *Pseudomonas*, within the *P. aeruginosa* lineage [28] with greatest similarity to “Pseudomonas azelaica” DSM 9128^T^ (99%), *Pseudomonas nitroreducens* DSM 14399^T^ (98%: *Pseudomonas multiresinivorans* ATCC 700690^T^ a heterotypic synonym) and *Pseudomonas jinjuensis* LMG 21316^T^ (98%), suggesting that strain Ind01 lies within one of those characterized species. However, comparison of key biochemical parameters identified a number of differences with respect to nutrient utilization between isolate Ind01 and these two strains (Table S1).

### Acephate utilization by *Pseudomonas sp.* Ind01 as a sole source of carbon

**Figure 2:**
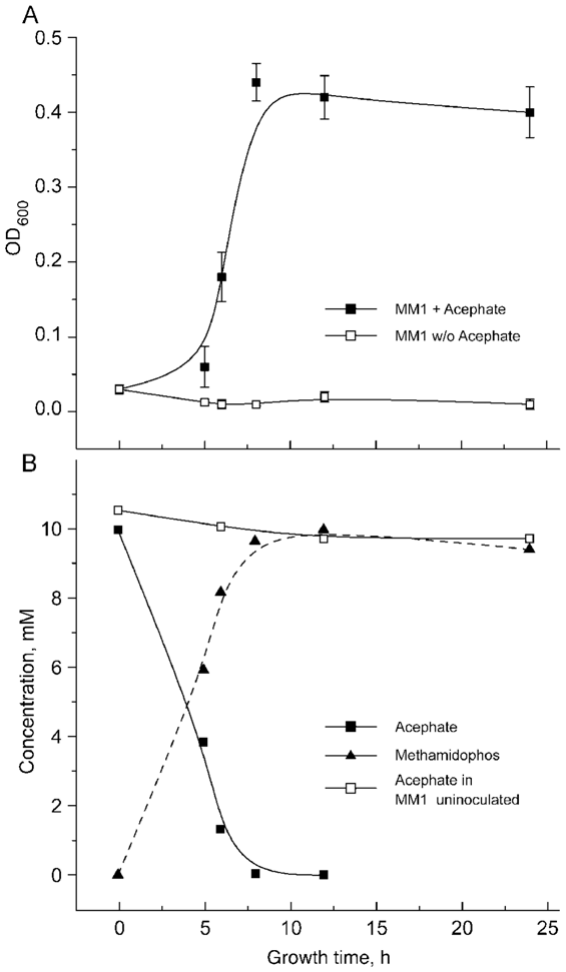
Growth kinetics and acephate utilization by *Pseudomonas* sp. Ind01 on MM1 growth media. (a) Growth kinetics monitored at λ 600 nm on MM1 supplemented with acephate (black squares) or w/o acephate (white squares) (b) Concentration of acephate (black squares) and methamidophos (black triangles) in MM1 medium, supplemented with 10 mM acephate as a sole source of carbon. Acephate concentration in uninoculated medium represented by white squares.

The ability of *the Pseudomonas sp.* Ind01 to utilize acephate as a sole carbon source was demonstrated by strain growth on MM1 supplemented with acephate, achieving a maximal growth rate 0.11 OD_600_/h. The same medium without acephate did not support growth (Fig. 2a). A time course analysis of the culture supernatant by HPLC determined that the increase in cell density was accompanied by a concomitant decrease in acephate concentration. As acephate was depleted, cell growth slowed and reached a stationary cell density, ultimately demonstrating the decline in density associated with cell death. At the same time, the concentration of the metabolite methamidophos increased through the first 5–6 hours of growth. Upon reaching its maximum, the concentration did not change during further incubation (Fig. 2b). The identity of the HPLC peaks was confirmed by GC-MS and LC-MS/MS analysis.

**Figure 3:**
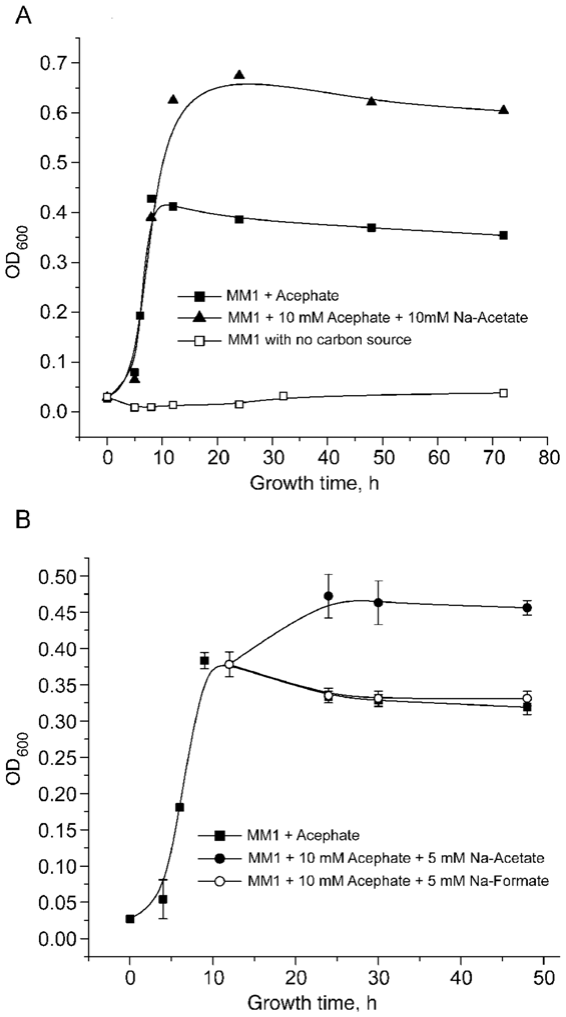
Growth kinetics of *Pseudomonas sp.* Ind01 on MM1 growth media supplemented with additional carbon source. (a) Growth of Ind01 on MM1 medium supplemented with 10 mM acephate (black squares), 10 mM acephate and 10 mM sodium acetate (black triangles) or with no carbon source (white squares) (b) Growth of Ind01 on MM1 medium supplemented with 10 mM acephate (black squares). After culture reached stationary phase, 5 mM sodium acetate (black circles) or 5 mM sodium formate (white circles) was added as an additional carbon sources. Each data point is an average value and error bars represent the standard deviation (n = 2).

An additional source of carbon (sodium acetate), delivered either prior to inoculation (Fig. 3a) or after the culture reached stationary phase (Fig. 3b), significantly enhanced culture growth, confirming that growth was limited by depletion of acephate as an available carbon source. Methanol (1.0%) or sodium formate (5 mM) was not able to supplement growth. *Pseudomonas sp.* Ind01 was able to grow on acephate at concentrations as high as 80 mM; maximum growth was observed at 40 mM (data not shown).

**Figure 4:**
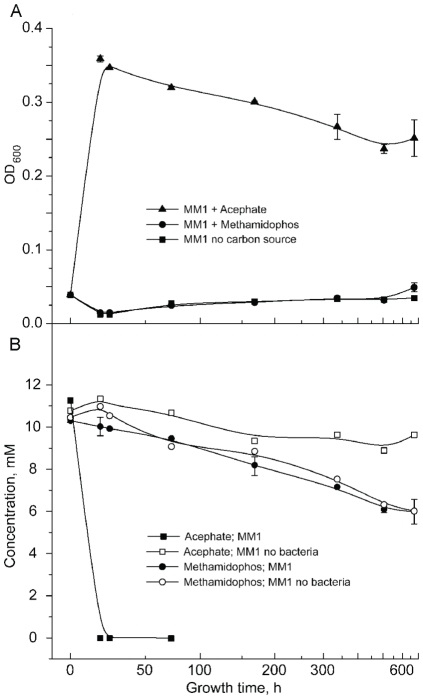
Growth kinetics of *Pseudomonas sp.* Ind01 on MM1 medium supplemented with acephate or methamidophos as a sole source of carbon. (a) Growth kinetics on MM1 medium, supplemented with 10 mM acephate (triangles), 10 mM methamidophos (circles), or without any carbon source (squares). (b) Concentration methamidophos (circles) and acephate (squares) in uninoculated (white) or inoculated (black) media. Average values and standard deviation (n = 2) are shown. Growth time is represented on an offset reciprocal scale.

Since there was no detectable decrease in the concentration of methamidophos generated during growth on acephate, it appeared that utilization of acephate was limited to a single degradative step. To verify this, MM1was supplemented with methamidophos as the sole carbon source. In contrast to acephate supplemented media, where the culture optical density increased at a rate of 0.11 OD/hr, there was no detectable growth on methamidophos supplemented MM1 media (Fig. 4a). In spite of this, approximately 40% of the provided methamidophos was depleted over the four-week incubation. However, the depletion of methamidophos from the inoculated medium and the uninoculated control had very similar profiles (Fig. 4b), supporting an argument that the breakdown of methamidophos was abiotic.

### Characterization of growth on acephate as a source of sulphur or nitrogen

**Figure 5:**
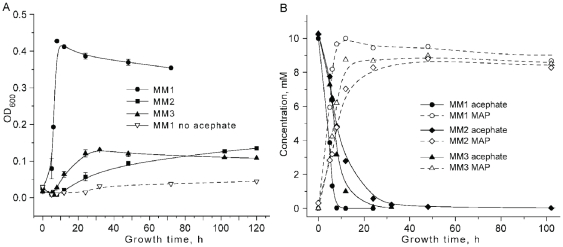
Growth kinetics of *Pseudomonas sp.* Ind01 on acephate as a sole source of C, S and N. (a) Growth kinetics on MM1 (white squares), MM2 (black circles) and MM3 (black triangle) media, supplemented with 10 mM acephate. Growth on control MM1 medium w/o acephate is represented by open triangles. (b) Acephate degradation (solid lines, black marks) and methamidophos accumulation (dashed lines, white marks) during growth on MM1 (circles), MM2 (triangles) and MM3 (diamonds) media, supplemented with 10 mM acephate. The concentration of acephate in control uninoculated MM1 medium is represented by black squares. Each data point represents the average values and error bars are the standard deviation (n = 3).

The molar ratio of C∶ N∶ S per mole of acephate is 4∶ 1∶ 1, so acephate potentially can serve as a source of nitrogen and sulphur, as well as carbon (Fig. 1). To assess this, *Pseudomonas sp.* Ind01 was inoculated into MM2 and MM3, supplemented with 10 mM acephate. Control cultures included MM1 medium supplemented with 10 mM acephate as a carbon source, as well as inoculated MM1 media with no supplements and each supplemented medium without an inoculum. Although the isolate demonstrated limited growth on MM2 and MM3, medium in which acephate provided the sole C and N or S source, the growth rate was significantly lower than on acephate as a C source: 0.11 ΔOD_600_/h for MM1 and 0.003 and 0.01 ΔOD_600_/h for MM2 and MM3, respectively (Fig. 5a). In contrast to growth on MM1, the addition of sodium acetate to the MM2 and MM3 media did not enhance the growth (data not shown), confirming that C was not the limiting nutrient, but that growth was indeed limited by the available nitrogen and sulphur. The rates of acephate degradation and accumulation of methamidophos in MM2 and MM3 media were reduced relative to MM1 medium, corresponding to the reduced growth rates on those media (Fig. 5b). The degradation of acephate can be described by a first order equation of exponential decay with a half-life for acephate of 5 h, 8 h, and 11.5 h on MM1, MM3, MM2 media, respectively.

### Resting cell assays suggest a role for methamidophos as a sulphur donor for growth

A resting cell assay analyzed by GC-MS and LC-MS/MS was employed to identify catabolic intermediates of acephate degradation. When metabolites extracted from the spent medium were analyzed by GC-MS, two peaks were observed. Under the chromatographic conditions described, these peaks were found to have retention times of 4.0 and 13.0 minutes, respectively, and mass spectral analysis determined them to have identical spectral properties as that of acetic acid (molecular ion M^+^ at *m/z* 60) and methamidophos (molecular ion M^+^ at *m/z* 141). This is consistent with the previously proposed pathway in which acephate degradation is initiated through a hydrolytic cleavage of the amide linkage found between these two moieties. (Fig. 1)

**Figure 6:**
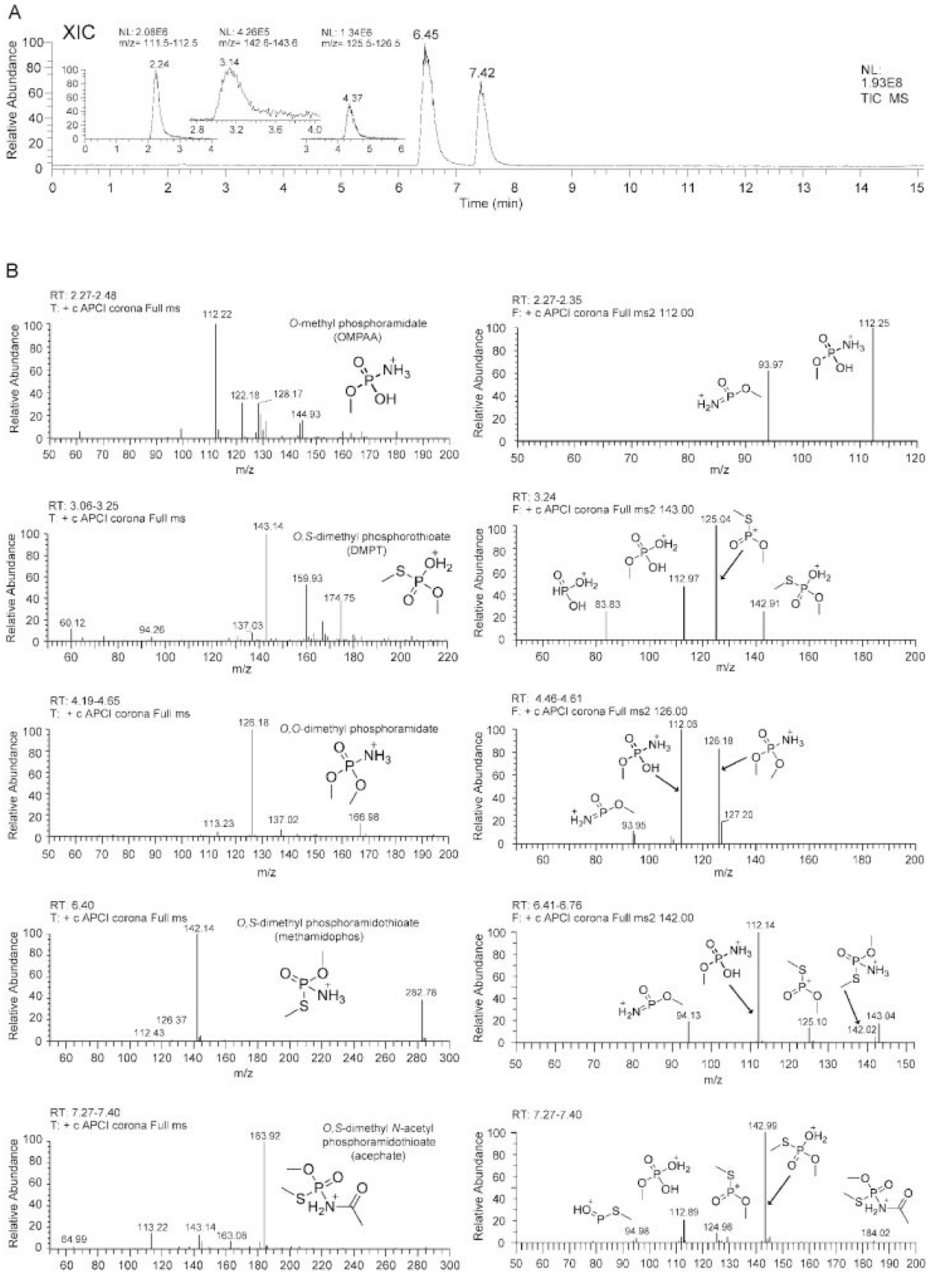
Identification of degradation products of acephate degradation by LC-MS/MS. (a) Total ion counting chromatogram of acephate degradation products after 30 min incubation in Resting Cell Assay and extracted ion counting chromatogram fragments for [M+H]+m/z 112, m/z 126 and m/z 143 ions after 96 h incubation in RCA (insert). (b) Mass spectra of the peaks observed at retention time 2.2, 3.1, 4.4, 6.4, 7.4 and min in a left and MS/MS fragmentation patterns of main compounds of the corresponding peaks in a right column.

Owing to their high solubility in water, reverse-phase HPLC and LC-MS were used for the identification of potential degradation products. Two main peaks with retention times of 7.4 and 6.4 min were detected, which were correspondingly identified as acephate ([M+H]^+^ at *m/z* 184) and methamidophos ([M+H]^+^ at *m/z* 142) (Fig. 6). A dimer of the protonated methamidophos was also detected at *m/z* 283. In addition to acephate and methamidophos, three other compounds characterized by protonated molecules [M+H]^+^ at *m/z* 112 (retention time 2.2 min), *m/z* 143 (retention time 3.1 min) and *m/z* 126 (retention time 4.3 min) were detected. The retention time (2.2 min) is close to the column dead time and hence impurities (peak other than *m/z* 112) were also observed in the mass spectrum. In addition, oxidation of the protonated molecule at *m/*z 143 (retention time 3.1 min) occurred during the ionization process resulting in successive increase of *m/z* by 16 Da from *m/z* 143 to 159 and 175. The MS/MS of each protonated molecule is shown in Fig. 6b to the left of each mass spectrum and the chemical structures of the fragment ions are indicated in each mass spectrum to confirm the identity of these compounds. Isotopic distribution of each protonated molecule was also considered for identification. Out of the three compounds, only the compound at retention time 3.1 (*m/z* 143) was shown to contain sulphur. These three compounds at retention times 2.2, 3.1 and 4.3 min were identified as *O,S*-dimethyl phosphorothioate (*m/z* 143) *O,O*-dimethyl phosphoramidate (*m/z* 126) and *O*-methyl phosphoramidate (*m/z* 112) respectively. The peak areas of these compounds never exceeded 1–2% relative to acephate and methamidophos peak areas (Fig. 6a), and they were not detected in the control cell suspension without substrate, eliminating the biomass as a source.

**Figure 7:**
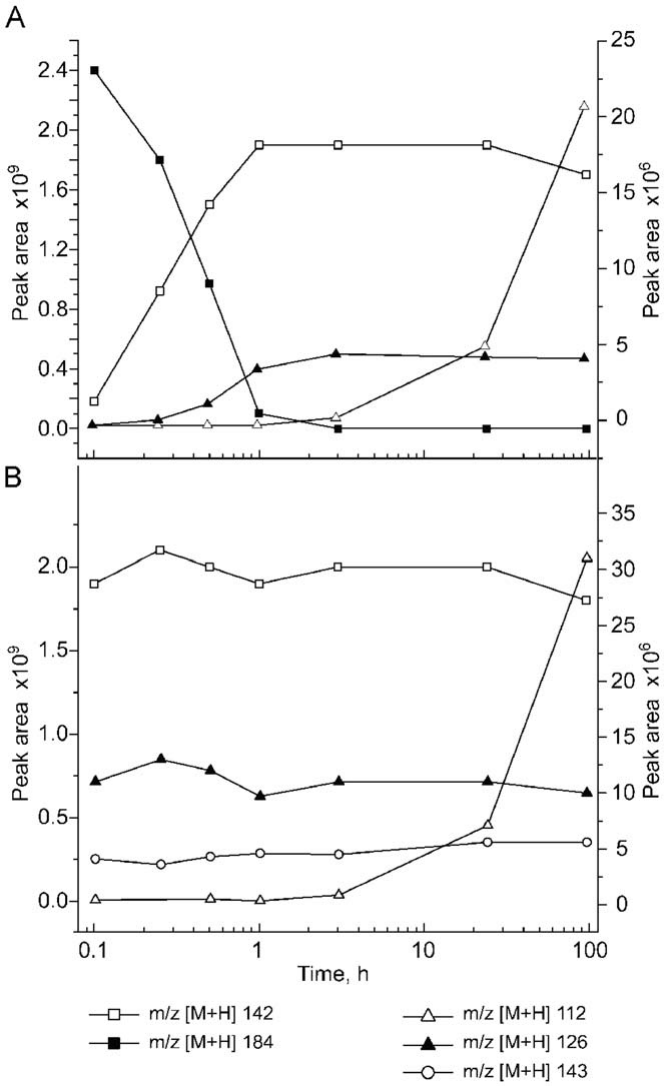
Kinetics of metabolites during acephate (A) or methamidophos (B) degradation in Resting Cell Assay. Left Y-axis: [M+H]^+^ ions m/z 184, acephate; m/z 142, methamidophos; right axis: [M+H]^+^ ions m/z 126, *O,O*-dimethyl phosphoramidate; m/z 112 *O*-methyl phosphoramidate and m/z 143, *O,S*-dimethyl phosphorothioate.

Acephate is almost completely degraded within one hour (Fig. 7). Over the same timeframe, methamidophos increased to its maxima, where it maintained a relatively stable concentration over 96 h. Quantification with HPLC analysis confirmed that the concentration of acephate decreased from 10 mM to undetectable levels, while methamidophos increased to an approximately equimolar amount (above 9.3 mmol) over the same period (data not shown). They appear to be products of methamidophos degradation or decomposition, although they have distinctly different accumulation kinetics.

*O*-methyl phosphoramidate (*m/z* 112) was first detected shortly after methamidophos reached a maximum and then accumulated throughout the remaining assay time course (Fig. 7). This compound can be generated as a result of methamidophos decomposition with the release of methyl mercaptan (Fig. 1). Importantly, virtually identical accumulation kinetics was observed in control tubes without cells (data not shown), demonstrating its abiotic origin.

In the acephate RCA, *O,O*-dimethyl phosporamidate (*m/z* 126) accumulated with kinetics similar to methamidophos, although with a longer lag phase which resulted in a delayed time to maximum (Fig. 7a). The negative control without cells did not generate this compound. In the methamidophos RCA, there was a small amount present at the zero time point and its concentration remained virtually constant throughout the assay (Fig. 7b). This was observed in both the RCA reaction, as well as in the negative control without cells.

Similar kinetics were observed for *O,S*-dimethyl phosphorothioate (*m/z* 143) in the methamidophos RCA. A small amount was present at time 0 and remained unchanged throughout the assay, in both the RCA reaction as well in the negative control without cells (Fig. 7b). It was only detected in trace amounts in the acephate RCA.

## Discussion

Acephate has been extensively used as an insecticide to control a number of agriculturally important insect pests and, in spite of a relatively short half-life, it has been found in various components of the environment [3]. Although there are extensive studies on the biodegradation of various other organophosphates, only a few reports are available on bacterial-promoted biodegradation of acephate [2], [29], [30], [31].

It was shown by early studies that acephate degraded in soils and water through methamidophos or *O*-methyl N-acetylphosphoramidate (Fig. 1). The identification of these two metabolites suggested two alternative degradation pathways. The generation of *O*-methyl N-acetylphosphoramidate would require a phosphotriesterase-type enzyme to hydrolyze the P-S bond [32], [33], while methamidophos would be generated by a carboxylesterase-type enzyme, releasing the acetate residue. Although there have been several reported studies focused on the methamidophos biodegradation pathway, the details of acephate biodegradation have still not been elucidated. In the present study, the isolation of acephate-degrading bacterium from activated sludge from a pesticide manufacturing unit and some insight into acephate degradation is reported.

Based on the phenotypic and phylogenetic analysis, the isolate is designated as *Pseudomonas sp.* Ind01. The bacterium is capable of hydrolyzing acephate to methamidophos and acetic acid, which is used as a source of carbon. Surprisingly, *Pseudomonas sp.* Ind01 failed to use or co-metabolize other tested OP compounds, such as paraoxon and parathion. Consistent with this phenotype, neither OPH activity nor an *opd* gene was detected in this strain (data not show). In addition, the strain is not able to utilize the tested C1 compounds (methanol and sodium formate) as a sole carbon source (Table S1, Fig. 3), in contrast to ethanol, which could support growth.

Potentially, acephate can also serve as a source of nitrogen and sulphur (Fig. 1). Indeed, a number of soil microbes, such as *Penicillium* [34], *Hyphomicrobium* and *Luteibacter* [35] have been reported to be able to utilize methamidophos, a first degradation product in acephate mineralization, as a sole source of nitrogen or sulphur. Our data does not provide evidence that the *Pseudomonas sp.* Ind01 is able to mineralize methamidophos. Although methamidophos as a sole carbon source did not support growth on MM1 medium (Fig. 4a), a limited growth on carbon and sulphur or nitrogen deficient media (MM2 and MM3, respectively), supplemented with acephate, was observed (Fig. 5a). This growth may be supported by abiotic hydrolysis of methamidophos in water solution to *O*-methyl phosphoramidate with the corresponding release of methyl mercaptan, which has been shown to be utilized as a sulphur source by bacteria, including some *Pseudomonas* [36], [37]. The accumulation of *O*-methyl phosphoramidate ([M+H]^+^ m/z 112), observed in the resting cell assay supports this hypothesis (Fig. 7). Similarly, the abiotic generation of *O,S*-dimethyl phosphorothioate can provide sufficient nitrogen for such limited growth.

It has been reported that the metabolism of some pesticides may by significantly enhanced in the presence of an additional carbon source, i.e. the degradation of chlorpyrifos by *Enterobacter* in the presence of succinate [38]. In this study, the addition of sodium acetate or glucose, either at the time of inoculation or after the culture reached stationary phase, did not result in enhancement of methamidophos degradation. On the other hand, no inhibition in the acephate degradation rate was observed when cells were grown on a mixture of acephate and sodium acetate or dextrose, indicating that the acephate-degrading system is not activated (de-repressed) under carbon starvation (data not shown). The inability to grow on methamidophos as a sole carbon source is consistent with the inability of the strain to grow on C1 compounds, such as methanol and sodium formate (Table S1; Fig. 3, 4) and suggests that the strain does not possess the enzyme systems needed for either the extraction or utilization of methyl groups. Strains such as described in this work can, however, promote the first step of acephate mineralization in a soil microbial community.

Acephate has been shown to have potential to contaminate ground water [20], [21], [22]. In a typical Ground Water Pollution-potential model (GWP), acephate has reached ground water after leaching out from a variety of soils [22]. However, due to higher absorption coefficient, methamidophos is shown to remain in most of the soil types. In such a scenario presence of *Pseudomonas* sp. Ind01 might help in converting otherwise penetrative acephate to a more absorbable methamidophos. If this is seen together with the existence of methamidophos degrading soil microflora [27], [35], the true potential of *Pseudomonas* sp. Ind01 to remediate acephate contaminated soils can easily be realized.

## Methods

### Isolation and characterization of acephate-degrading bacterium

For isolation and characterization of the acephate-degrading bacterium, minimal media lacking a source of carbon (MM1), carbon and nitrogen (MM2) and carbon and sulphur (MM3) were used (Table 1). All medium salts were no less than ACS or GR grade, with a nitrogen and sulphur content less than 0.002%. Acephate (*O,S*-dimethyl acetylphosphoramidothioate) and methamidophos (*O,S*-dimethyl phosphoramidothioate) were purchased from ChemService Inc. (West Chester, PA). For DNA purification and propagation of stock cultures for growth experiments, cells were grown on LB medium supplemented with 50 µg/ml carbenicillin.

**Table 1:** Composition of minimal media used for isolation and characterization of acephate degrading bacterium.

		g/L	
	MM1 [C]	MM2 [C-N-]	MM3 [C-S-]
K_2_HPO_4_	4.8	4.8	4.8
KH_2_PO_4_	1.2	1.2	1.2
MgSO_4_.7H_2_O	0.2	0.2	-
FeSO_4_.7H_2_O	0.001	0.001	-
NH_4_NO_3_	1	-	1
Ca(NO_3_)_2_. 4H_2_O	0.04	-	0.04

An acephate-degrading bacterium was isolated through an enrichment culture technique using activated sludge collected from a pesticide-manufacturing unit (Rallis India Ltd, Patancheru, Hyderabad, Andhra Pradesh, India). A 250 ml conical flask containing 50 ml minimal medium MM1 supplemented with 0.5 mM acephate as a sole source of carbon was inoculated with 10 g of activated sludge. The flask was incubated at 30°C on an orbital shaker at 200 rpm for a period of 7 days. After the incubation period, the sludge material was allowed to settle and 1 ml of particulate free suspension was re-inoculated into a fresh 50 ml of MM1 medium supplemented with acephate (0.5 mM). Four such transfers were made and during each transfer the enriched population was plated on MM1 medium supplemented with acephate as a source of carbon. After the fourth transfer, a pure culture capable of growing on acephate was obtained and designated strain Ind01 before depositing in DSMZ, Germany with an accession number DSM 19477.

### Phenotypic and Phylogenetic Characterization

Genomic DNA was extracted and purified using the Qiagen DNeasy Blood and Tissue kit (Qiagen Ink., Valencia, CA). PCR amplification of the 16 S rRNA gene and its complete sequence was obtained following procedures described elsewhere [39], [40]. The sequence of 16 S rRNA gene of strain Ind01 is deposited in NCBI data base (AM407893). The CLUSTAL_W algorithm of MEGA 4 was used for sequence alignments and MEGA 4 [41] software was used for phylogenetic analyses of both individual and concatenated sequences. Distances were calculated by using the Jukes and Cantor correction in a pair wise deletion procedure. Unweighted pair group with mathematical average (UPGMA), neighbour-joining (NJ), minimum evolution (ME) and maximum parsimony (MP) methods in the MEGA4 software were used to construct phylogenetic trees. Percentage support values were obtained using a bootstrap procedure.

Biochemical growth properties were assessed by growing the strain Ind01 in MM1 medium supplemented with carbon sources (Table S1) to a final concentration of 0.3% (w/v). Growth was monitored spectrophotometrically (at OD_600_) for a period of 72 h. While testing for antibiotic sensitivity, the strain was plated independently on LB plates having chloramphenicol (30 µg/ml), ampicillin (100 µg/ml) or kanamycin (25 µg/ml).

### Growth studies with acephate as a sole source of carbon, nitrogen and sulphur

After obtaining a pure culture, the strain's ability to use acephate as a source of carbon, nitrogen or sulphur was assessed by growth in minimal medium lacking alternative sources for each essential element. Acephate (10 mM) was added to a minimal medium formulated to be free of alternative carbon (MM1), carbon and nitrogen (MM2) or carbon and sulphur (MM3). Sodium acetate (1–10 mM), sodium formate (5 mM), dextrose (5 mM) or ammonium chloride (0.1–0.5 mM) were used in some experiments as an additional carbon or nitrogen source, respectively.

For growth studies, LB medium was inoculated with colonies from a carbon-minus MM1 plate supplemented with 10 mM acephate and incubated overnight (11–16 h) at 30°C and 300 rpm. After overnight growth, the cells were collected by centrifugation (20 min at 3600×*g*), washed and resuspended in sterile water (OD_600_∼2). This stock suspension was inoculated into each of the minimal medium formulations (dilution ratio 1∶100) supplemented with 10 mM acephate and allowed to grow at 30°C and 300 rpm. The MM1 medium without acephate and the same medium supplemented with acephate without inoculum served as controls. At defined time points, sample aliquots were withdrawn, OD_600_ was recorded and cells were removed by centrifugation for 2 min at 14, 000×*g*. Supernatants were stored at −20°C until analyzed. Growth rate was estimated by the slope of the line representing the linear fit of the increase in optical density over time, during the exponential phase of culture growth.

### Resting cell assay (RCA)

A resting cell suspension (OD_600_ = 2.1) was created by growing strain Ind01 overnight at 30°C and 300 rpm (MM1 medium with acephate as the sole carbon source), harvested by centrifugation (20 min at 3600×*g*), washed twice with 15 ml of sterile citrate saline buffer (20 mM citrate buffer, pH 7.0, 150 mM NaCl) and then resuspended in either 18 ohm water or carbon minus MM1 medium. Filter sterilized acephate was added to each cell suspension up to a final concentration of 10 mM. Suspensions were then incubated at room temperature; samples (100 µl) were collected at 0, 5, 15, 30, 60, 90 min, 18 h and 96 h. Cells were removed by a 2 min centrifugation at 14,000×*g*. The cleared supernatant was stored at −20°C until analyzed.

### HPLC analysis

Reverse-phase HPLC (RP-HPLC) was used to monitor acephate degradation. Cleared supernatants from either growth experiments or RCA were diluted 1∶1 with 0.1% formic acid and analyzed using a Beckman Gold HPLC system equipped with an Aqua C18 column (250×4.6 mm, Phenomenex, Torrance, CA) and a diode-array detector. Acephate and metabolites were separated by gradient elution at 0.75 ml/min in a formic acid – methanol solvent system (eluent A: 0.1% formic acid in water, eluent B: 100% methanol); peak elution was monitored at 220 nm. The following gradient was used: t = 0, 0% B; t = 3.6 min, 50% B; t = 12 min, 80% B; t = 15.6 min, 100% B, followed by a column wash with 100% B and re-equilibration with 0.1% formic acid. For quantification of acephate and methamidophos, a calibration curve was obtained using commercially available standards (ChemService, West Chester, PA). Standards were not available for other metabolites.

### LC-MS analysis

Water-soluble metabolites were analyzed using a Surveyor HPLC system (Thermo Finnigan, San Jose, CA) interfaced with quadrupole ion trap mass spectrometer (LCQ-DECA; Thermo Electron). Separation was performed using an Aquasil C18 reversed phase column (2.1 mm×150 mm, 3 µm) (Thermo Electron, San Jose, CA). The gradient mobile phase consisted of water (A) and methanol (B), each containing 0.1% formic acid. At a flow rate of 0.2 ml/min, a gradient was started at 0% B, increased to 60% in 5 minutes and then to 100% in 3 min where it was held for 2 min prior to being returned to 0% B in 0.5 min. The solvent system was held at 0% B for 5 min to re-equilibrate the column between runs.

An APCI probe in the positive ion mode was used for ionization. The MS operating conditions were optimized as follows: sheath gas and auxiliary gas flow rate, 50 and 10 arbitrary units, respectively (both high purity nitrogen), APCI vaporizer temperature, 450°C, corona current, 5 µA. Transfer capillary temperature was held at 150°C. Relative collision energy of 30% was used for collision-induced dissociation. Xcalibur 2.0 (Thermo Finnigan) was used to control the LC-MS system and for data acquisition and processing.

### GC-MS analysis

For GC-MS analysis, 50 ml of the spent medium was clarified by centrifugation at 13,200 *g*, followed by filtration through a 0.2 µm filter. The clarified medium was acidified with 0.09 N hydrochloric acid and then extracted thrice with an equal volume of ethyl acetate. The pooled organic phase was allowed to air dry, and the remaining residue was dissolved in a minimal volume (500 µl) of methanol and about 1 µl of it was taken for analysis using a GC-MSQP5050A (Shimadzu) equipped with a column (25 m×0.2 mm ID×0.33 µm) packed with 100% SPBI (Supelco). The column, injection port and detector temperatures were maintained at 210°C, 230°C and 250°C, respectively. Helium was used as the carrier gas and the flow rate was maintained at 1 ml/min.

## Supporting Information

Figure S1 **Cell morphology of acephate degrading strain Ind01.**
(TIFF)

Figure S2 **Dendrogram of the phylogenetic relationships based on 16 S rRNA gene analysis.** The scale bar corresponds to 2 nucleotide substitutions per 100 nucleotides. Numbers indicate statistical significance of the branching order determined using bootstrap analysis of 100 alternative trees; dendrogram was constructed using MEGA 4.1.
(TIF)

Table S1 Comparison of biochemical properties of acephate degrading strain Ind01 and type strains.* ^*^Data for *P. azelaica*, *P. nitroreducens* and *P.citronelosis* and *P. multiresinivorans* taken from or consistent with Lang et al. (Lang, E., Griese, B., Sproer, C., Schumann, P., Steffen, M. &Verbarg, S. (2007). Characterization of ‘*Pseudomonas azelaica*’ DSM 9128, leading to emended descriptions of *Pseudomonas citronellolis* Seubert 1960 (Approved Lists 1980) and *Pseudomonas nitroreducens* Iizuka and Komagata 1964 (Approved Lists 1980), including *Pseudomonas multiresinivorans* as its later heterotypic synonym. Int J Syst Evol Microbiol 57, 878–882); data for *P.jinjuensis* taken from Kwon et al. (Kwon, S. W., Kim, J. S., Park, I. C., Yoon, S. H., Park, D. H., Lim, C. K. & Go, S. J. (2003). *Pseudomonas koreensis* sp. nov., *Pseudomonas umsongensis* sp. nov., and *Pseudomonas jinjuensis* sp. nov., novel species from farm soils in Korea. (Int J Syst Evol Microbiol 53, 21–27.).
(DOC)
