# Supplementary figures and images for: Mineralization of Acephate, a Recalcitrant Organophosphate Insecticide Is Initiated by a Pseudomonad in Environmental Samples
Source: PLoS One. 2012 Apr 4;7(4):e31963. doi: 10.1371/journal.pone.0031963 (PMC3319554; doi:10.1371/journal.pone.0031963)

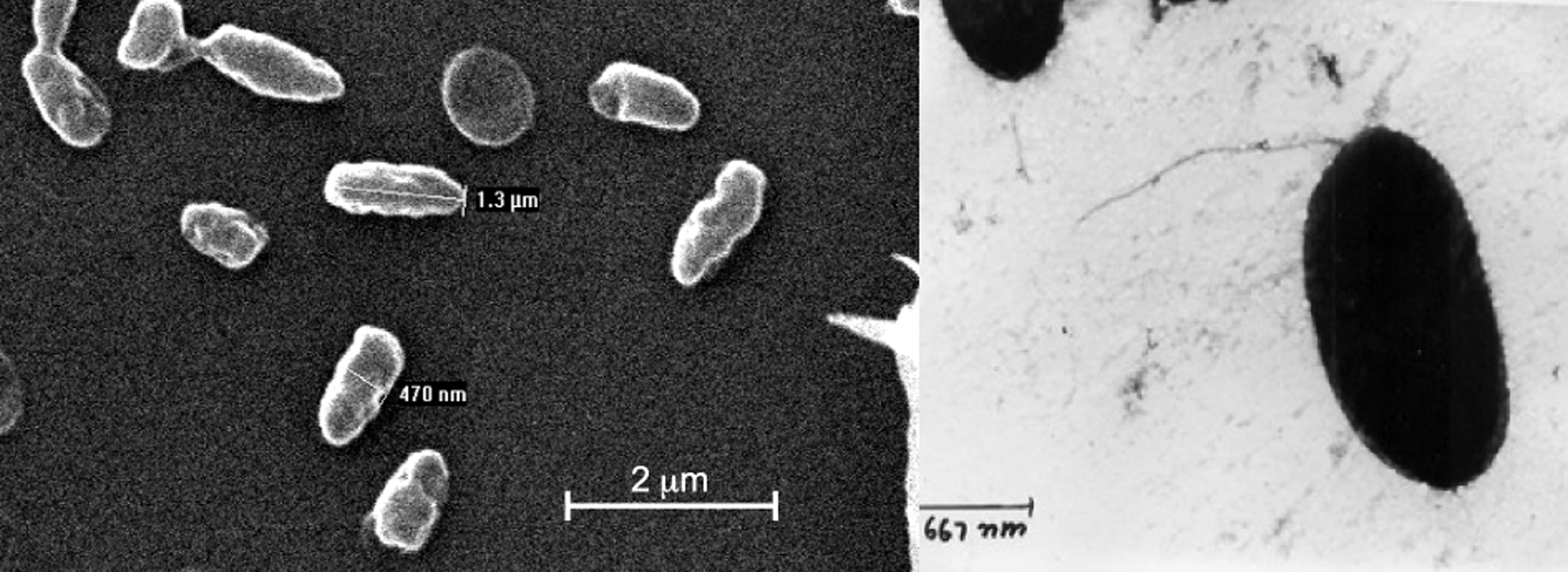

Supplement: Figure S1 — Cell morphology of acephate degrading strain Ind01. (TIFF) [file pone.0031963.s001.tif]

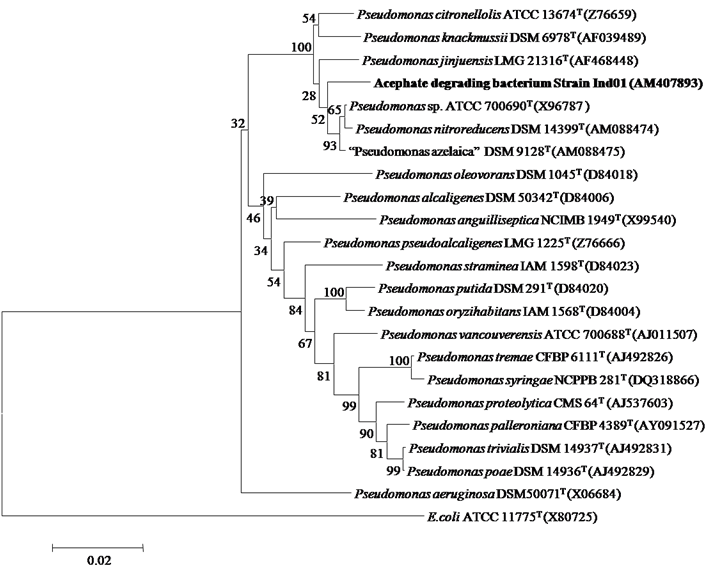

Supplement: Figure S2 — Dendrogram of the phylogenetic relationships based on 16 S rRNA gene analysis. The scale bar corresponds to 2 nucleotide substitutions per 100 nucleotides. Numbers indicate statistical significance of the branching order determined using bootstrap analysis of 100 alternative trees; dendrogram was constructed using MEGA 4.1. (TIF) [file pone.0031963.s002.tif]
