# Supplementary material for: Mineralization of Acephate, a Recalcitrant Organophosphate Insecticide Is Initiated by a Pseudomonad in Environmental Samples
Source: PLoS One. 2012 Apr 4;7(4):e31963. doi: 10.1371/journal.pone.0031963 (PMC3319554; doi:10.1371/journal.pone.0031963)
Supplement: Table S1 — Comparison of biochemical properties of acephate degrading strain Ind01 and type strains.* *Data for P. azelaica, P. nitroreducens and P.citronelosis and P. multiresinivorans taken from or consistent with Lang et al. (Lang, E., Griese, B., Sproer, C., Schumann, P., Steffen, M. &Verbarg, S. (2007). Characterization of ‘Pseudomonas azelaica’ DSM 9128, leading to emended descriptions of Pseudomonas citronellolis Seubert 1960 (Approved Lists 1980) and Pseudomonas nitroreducens Iizuka and Komagata 1964 (Approved Lists 1980), including Pseudomonas multiresinivorans as its later heterotypic synonym. Int J Syst Evol Microbiol 57, 878–882); data for P.jinjuensis taken from Kwon et al. (Kwon, S. W., Kim, J. S., Park, I. C., Yoon, S. H., Park, D. H., Lim, C. K. & Go, S. J. (2003). Pseudomonas koreensis sp. nov., Pseudomonas umsongensis sp. nov., and Pseudomonas jinjuensis sp. nov., novel species from farm soils in Korea. (Int J Syst Evol Microbiol 53, 21–27.). (DOC) [file pone.0031963.s003.doc]

## Supplementary Table S1. Comparison of biochemical properties of acephate degrading strain Ind01 and type strains*

|  | Strain Ind01 | *P. azelaica*  DSM 9128T | *P. nitroreducens*  DSM 14399T | | *P. citranellolis*  DSM 50332T | *P. multiresinivorans*  DSM 700690T | *P. knackmussii*  DSM 6978T | *P. jinjuensis*  LMG 21316T |
| --- | --- | --- | --- | --- | --- | --- | --- | --- |
| Utilization of carbon sources | | | | | | | | |
| Glucose | + | + | | + | + | + | + | + |
| Lactose | + | – | | – | – | – | – | – |
| Cellobiose | + | – | | – | – | – | – | – |
| Maltose | ? | – | | – | – | – |  | – |
| DL-Malate | + | + | | + | + | + | + | + |
| Anthranilate | – |  | |  |  |  |  |  |
| Salicylate | – | – | | – | – | – |  |  |
| Succinic acid | + | + | | + | + | + | + | + |
| Benzoic acid | + | + | | + | + | + | + | + |
| p-Aminobenzoic acid | + | – | | – | – | – | – | – |
| L-glutamic acid | + | + | | + | + | + | + | + |
| Sodium citrate | + | + | | + | + | + |  | + |
| Sodium acetate | + | w | | + | + | + |  | ± |
| Sodium formate | – | w | | + | + | + |  | + |
| K/Na tartrate^&^ | – | – | | – | – | – |  |  |
| Potassium gluconate | + | + | | + | + | + |  | + |
| Dulcitol | + | – | | – | – | – | – | – |
| Methanol | - |  | |  |  |  |  |  |
| Acetamide | + | – | | – | – | – |  |  |
| Ethanol | + | + | | + | + | + |  |  |
| Acephate | + | – | | – | – | – | – | – |
| Hydrolysis of starch | – | – | | – | – | – |  | – |
| Hydrolysis of gelatin | + | – | | – | – | – | – | – |
| Nitrate Reduction | + | + | | + | + | + |  | + |
| Acid Production | | | | | | | | |
| Dextrose | – | – | | – | – | – |  | – |
| Maltose | – |  | |  |  |  |  |  |
| Lactose | – |  | | – |  |  |  |  |
| Gas Production | | | | | | | | |
| Lactose | + | – | | – | – | – | – | – |
| Dulcitol | + | – | | – | – | – | – | – |
| Cellobiose | + | – | | – | – | – | – | – |
| Growth @37°C | + |  | | – |  |  |  |  |
| Growth @41°C | - | - | | w | + | w |  | + |

w – weak growth;

^*^Data for *P. azelaica*, *P. nitroreducens* and *P.citronelosis* and *P. multiresinivorans*

taken from or consistent with Lang et al. (Lang, E., Griese, B., Sproer, C., Schumann, P., Steffen, M. &Verbarg, S. (2007). Characterization of '*Pseudomonas azelaica*' DSM 9128, leading to emended descriptions of *Pseudomonas citronellolis* Seubert 1960 (Approved Lists 1980) and *Pseudomonas nitroreducens* Iizuka and Komagata 1964 (Approved Lists 1980), including *Pseudomonas multiresinivorans* as its later heterotypic synonym. Int J Syst Evol Microbiol 57, 878-882); data for *P.jinjuensis* taken from Kwon et al. (Kwon, S. W., Kim, J. S., Park, I. C., Yoon, S. H., Park, D. H., Lim, C. K. & Go, S. J. (2003). *Pseudomonas koreensis* sp. nov., *Pseudomonas umsongensis* sp. nov., and *Pseudomonas jinjuensis* sp. nov., novel species from farm soils in Korea. (Int J Syst EvolMicrobiol 53, 21-27.)
